# Opposing Shh and Fgf signals initiate nasotemporal patterning of the zebrafish retina

**DOI:** 10.1242/dev.125120

**Published:** 2015-11-15

**Authors:** María Hernández-Bejarano, Gaia Gestri, Lana Spawls, Francisco Nieto-López, Alexander Picker, Masazumi Tada, Michael Brand, Paola Bovolenta, Stephen W. Wilson, Florencia Cavodeassi

**Affiliations:** 1 Centro de Biología Molecular Severo Ochoa (CSIC-UAM), C/Nicolás Cabrera 1, 28049, Madrid, Spain; 2 Department of Cell and Developmental Biology, University College London, Gower Street, London WC1 6BT, UK; 3 Center of Regenerative Therapies Dresden (CRTD), Biotechnology Center, Dresden University of Technology, 01062 Dresden, Germany; 4 CIBER de Enfermedades Raras (CIBERER), C/Nicolás Cabrera 1, 28049, Madrid, Spain

**Keywords:** Retina, Nasotemporal patterning, Shh, Fgfs, Zebrafish

## Abstract

The earliest known determinants of retinal nasotemporal identity are the transcriptional regulators Foxg1, which is expressed in the prospective nasal optic vesicle, and Foxd1, which is expressed in the prospective temporal optic vesicle. Previous work has shown that, in zebrafish, Fgf signals from the dorsal forebrain and olfactory primordia are required to specify nasal identity in the dorsal, prospective nasal, optic vesicle. Here, we show that Hh signalling from the ventral forebrain is required for specification of temporal identity in the ventral optic vesicle and is sufficient to induce temporal character when activated in the prospective nasal retina. Consequently, the evaginating optic vesicles become partitioned into prospective nasal and temporal domains by the opposing actions of Fgfs and Shh emanating from dorsal and ventral domains of the forebrain primordium. In absence of Fgf activity, *foxd1* expression is established irrespective of levels of Hh signalling, indicating that the role of Shh in promoting *foxd1* expression is only required in the presence of Fgf activity. Once the spatially complementary expression of *foxd1* and *foxg1* is established, the boundary between expression domains is maintained by mutual repression between Foxd1 and Foxg1.

## Abstract (precis)

**Summary:** In the fish eye, Hh signalling from the ventral forebrain regulates spatial identity in the retina by promoting *foxd1* expression. This role is required only in the presence of Fgf activity.

## INTRODUCTION

**Fig. 1.**
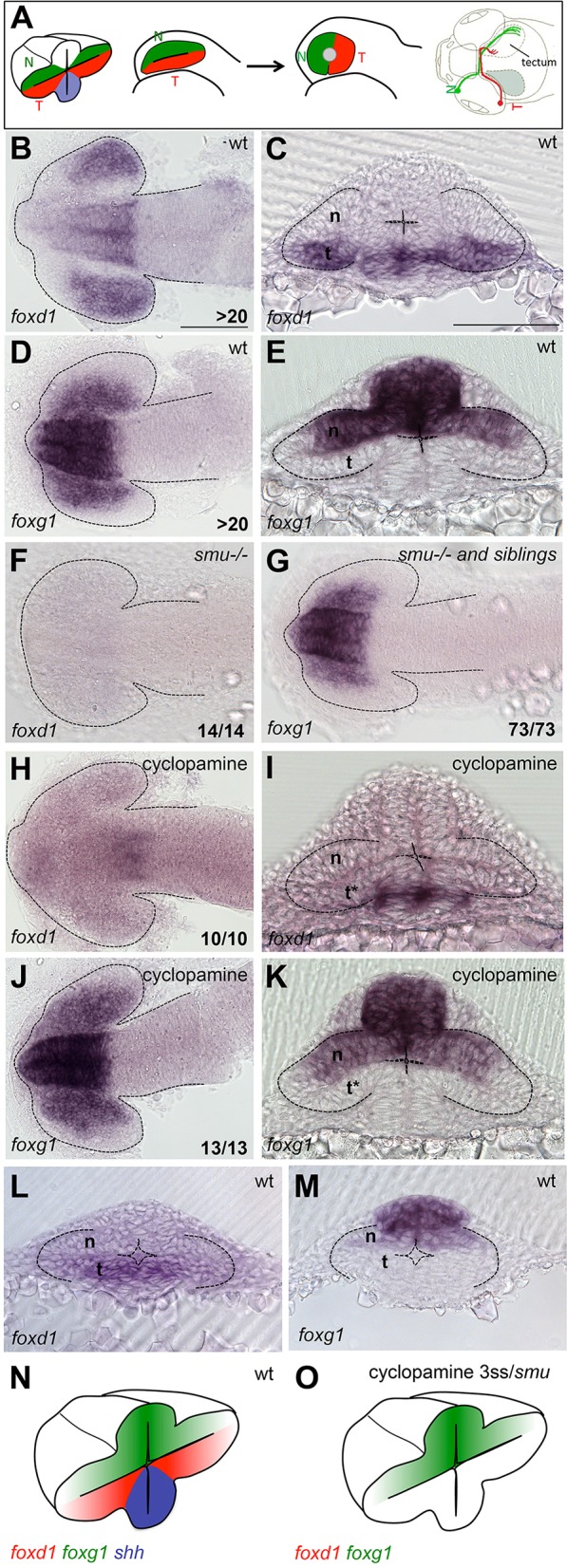
**Lack of Hh signalling results in loss of temporal fate.** (A) Schematic of early (left) and late (right) organisation of nasotemporal (NT) domains in the developing eye. Note that the NT axis, initially aligned with the DV axis of the embryo, rotates and becomes aligned with the AP axis as development proceeds. Red, temporal domain; green, nasal domain; blue, Shh source. (B-M) Dorsal with anterior to the left (B,D,F-H,J) and frontal (C,E,I,K-M) views of forebrain and eyes showing expression of *foxd1* (B,C,F,H,I,L) and *foxg1* (D,E,G,J,K,M) in the genotypes and treated conditions specified in the panels. All embryos were 10-12ss other than those shown in L and M, which were 5ss. (N,O) Schematic representations of the phenotypic outcome of *foxg1/foxd1* expression in wild-type (N) and lack of Shh (O) conditions. Scale bars: 100 µm. Numbers in the bottom-right of each panel indicate the number of embryos with the phenotype shown out of the total number of embryos analysed. n, nasal; t, temporal; t*, defective temporal domain. Dashed lines outline the forebrain (dorsal views) or the optic vesicles (frontal views).

Our ability to perceive the world around us and to represent visual information accurately requires correctly mapped innervation of the primary visual centres in the brain by retinal ganglion cell (RGC) axons. Map formation depends on the acquisition of specific positional identities by RGC precursors, as this information underlies the ability of RGC axons to connect appropriately within central targets (Erskine and Herrera, 2007; Schulte and Bumsted-O'Brien, 2008). The allocation of nasotemporal (NT) and dorsoventral (DV) positional identities in the eye primordium is already apparent at the optic vesicle stage, long before the first RGCs differentiate (Hatini et al., 1994; Picker et al., 2009). In fish, prospective retinal cells destined to form the nasal retina are initially located dorsally in the evaginating optic vesicle, whereas prospective temporal retina is located ventrally (Fig. 1A; Picker et al., 2009). A topologically similar organisation is probably present in other vertebrates with nasal retina originating next to dorsal telencephalic forebrain and temporal retina next to ventral, hypothalamic forebrain (Cobos et al., 2001).

The earliest known transcriptional determinants of NT identity are Foxg1 and Foxd1, which show complementary patterns of expression in prospective nasal and temporal domains of the eye primordium, respectively (Hatini et al., 1994). By a combination of loss- and gain-of-function approaches, *foxg1* has been shown to control cell proliferation and acquisition of nasal character during retinal patterning in mouse, chick, frog and zebrafish (Bourguignon et al., 1998; Hardcastle and Papalopulu, 2000; Huh et al., 1999; Martynoga et al., 2005; Picker et al., 2009). Complementarily, *foxd1* promotes acquisition of temporal character (Carreres et al., 2011; Herrera et al., 2004; Takahashi et al., 2009, 2003).

In zebrafish, genes encoding the Fgf ligands Fgf8, Fgf3 and Fgf24 are expressed in the forebrain and ectoderm dorsal to the evaginating optic vesicles, and collectively they promote *foxg1* expression and nasal identity in the dorsal optic vesicle (Picker and Brand, 2005; Picker et al., 2009). In the absence of Fgf activity, *foxg1* expression is lost, whereas, conversely, *foxg1* expands within the ventral half of the optic vesicle when the Fgf pathway is ectopically activated in this domain. The temporal determinant *foxd1* responds to Fgf activity in the opposite way. However, although *foxd1* expression expands into the dorsal optic vesicle in the absence of Fgfs, ectopic activation of Fgf activity in the ventral optic vesicle does not completely abrogate *foxd1* expression from this domain (Picker and Brand, 2005; Picker et al., 2009). These observations suggest that, in addition to Fgfs, other signals are involved in establishment of NT regionalisation and complementary *foxg1/foxd1* expression domains. In chick, for example, Wnt3a seems to modulate the expression of these genes, although a role for the Wnt pathway in controlling NT patterning has not been clearly demonstrated (Takahashi et al., 2009).

*shh* is expressed along the ventral midline of the forebrain in proximity to ventrally positioned, prospective temporal cells within the evaginating optic vesicles (Barth and Wilson, 1995; see also Fig. 3E). Shh is a morphogen and can generate a gradient of activity that confers different cellular identities according to the levels of ligand and the duration of the signal (Briscoe and Therond, 2013). Consequently, prospective temporal retinal cells may be exposed to Shh during the early phases of optic vesicle evagination and this pathway could therefore influence retinal NT patterning, together with Fgfs.

Although a role for Hh signalling in NT patterning has not been studied, this pathway does influence proximodistal (PD) regionalisation of the evaginated optic vesicle into optic stalk- and retina-forming territories (Ekker et al., 1995; Macdonald et al., 1995). Absence of Shh signalling is associated with loss of the optic stalk and cyclopia (Chiang et al., 1996; Macdonald et al., 1995; Varga et al., 2001). Conversely, excessive Shh signalling in the distal, prospective retinal portion of the optic vesicle interferes with retinal specification and promotes an expansion of proximal retinal and optic stalk fates (Cardozo et al., 2014; Ekker et al., 1995; Macdonald et al., 1995; Perron et al., 2003). Shh is also proposed to control DV regionalisation within the retina by promoting the expression of the ventral retinal determinant Vax2 (Lupo et al., 2005; Take-uchi et al., 2003).

In this study, we show that Shh activity is required to activate *foxd1* expression and to initiate temporal retinal identity at the onset of optic vesicle evagination in the zebrafish. Conditions in which Hh activity is lost result in the downregulation of *foxd1* expression. Conversely, ectopic Hh activity in the dorsal optic vesicle activates *foxd1* and represses *foxg1* in this domain. The changes in *foxg1/foxd1* expression upon activation of Hh signalling in evaginating optic vesicles result in altered NT retinal regionalisation and, as a consequence, abnormal targeting of retinal axonal projections in the tectum. Together with previous data, our study shows that NT patterning of the prospective retina is initiated in the optic vesicles by the opposing actions of the Fgf and Shh pathways. Although loss of Shh signalling leads to compromised specification of temporal identity and loss of Fgf signalling to compromised nasal identity, optic vesicles in which both pathways are blocked show recovery of *foxd1* expression, indicating that in the absence of Fgf activity the role of Shh in promoting temporal identity is dispensable. Overall, our results suggest that it is the appropriate balance between Shh and Fgf signals that ensures appropriate NT regionalisation in the forming eyes.

## RESULTS

### Abrogation of Hh signalling activity results in loss of temporal optic vesicle identity

At early stages of eye formation, cells destined to contribute to temporal retina are positioned ventrally as the optic vesicle evaginates from the forebrain (Fig. 1A). We hypothesised that signals emanating from ventral midline tissue of the forebrain may impart temporal character to prospective retinal cells. Among such candidate signals are Shh and Twhh (Shhb – Zebrafish Information Network), both of which are Hh signalling proteins expressed prominently in ventral forebrain tissue adjacent and ventral to the evaginating optic vesicles (Barth and Wilson, 1995; Ekker et al., 1995). Consequently, we assessed whether the expression of *foxd1*, the earliest known marker of prospective temporal retina is influenced by Hh signalling.

Abrogation of Hh activity in *smu* mutants (which lack function of the Smoothened Hh co-receptor; Varga et al., 2001) or in *syu* mutants (which lack Shh function; Schauerte et al., 1998) resulted in loss or downregulation, respectively, of *foxd1* expression in the ventral optic vesicle (Fig. 1B,F; data not shown). *foxg1* is normally expressed in prospective nasal retina in a spatially complementary pattern to *foxd1* (Fig. 1C,E). However, despite the absence of *foxd1* expression, *foxg1* expression did not expand into the ventral region of the optic vesicle in Hh pathway mutants (Fig. 1D,G). Together, these results suggest that initiation of temporal retinal identity requires Hh signalling but that acquisition of nasal identity requires more than just the absence of Hh activity.

Temporally controlled modulation of Hh activity using the Smo antagonist cyclopamine (Chen et al., 2002; Taipale et al., 2000) revealed that signalling is required in a narrow window at the start of optic vesicle evagination to promote *foxd1* expression. Although cyclopamine treatment starting at the 6-somite stage (ss) did not show any effect on *foxd1* expression or temporal fate specification (Fig. S1A-D), treatment from 1-3ss onwards resulted in a complete loss of *foxd1* expression (Fig. 1H,I) as well as of the *HGn42A::GFP* transgene (Picker et al., 2009), which specifically labels the temporal half of the eye primordium (Fig. S1E,F). Expression of the nasal markers *foxg1* and the *-8.0claudinb::lynGFP^zf106^* transgene (Haas and Gilmour, 2006) were not overtly affected by these treatments (Fig. 1J,K; Fig. S1I,J), a result consistent with the phenotype observed in *smu* and *syu* mutants. These results indicated that the Hh pathway is required between 1-3 and 6ss to promote temporal specification. At this stage, the optic vesicles are just starting to evaginate, but expression of *foxd1* and *foxg1* is already spatially restricted to complementary domains of the primordium (Fig. 1L,M).

### Ectopic Hh activity suppresses nasal and expands temporal identity in the optic vesicles

**Fig. 2.**
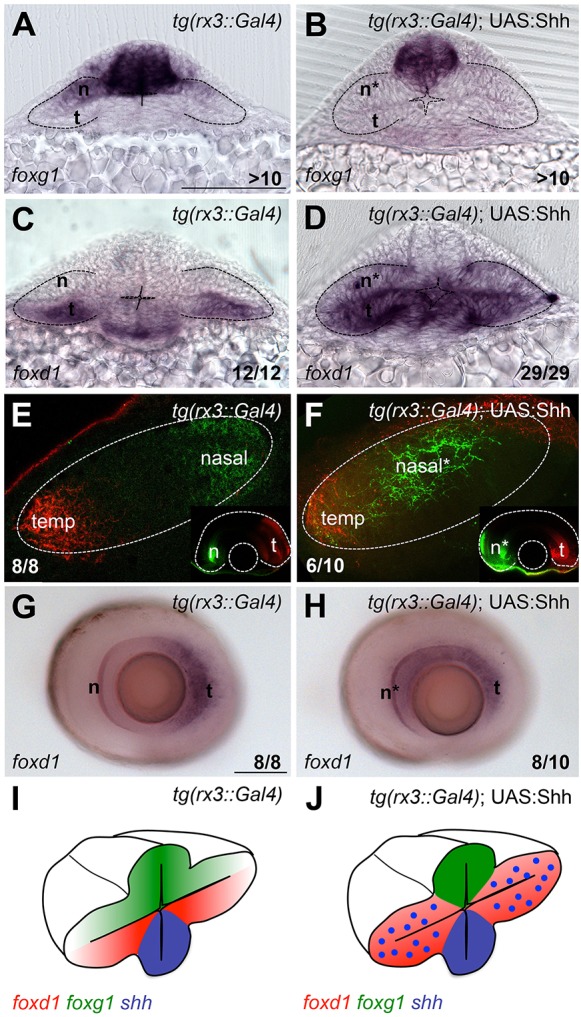
**Ectopic Hh activity in the optic vesicle promotes temporal fate.** (A-D,G,H) Expression of *foxg1* (A,B) and *foxd1* (C,D,G,H) in the genotypes specified in the panels. (E,F) Retinotectal projections traced with DiI/DiO to label nasal (n; green) and temporal (t; red) projections. Main panels show the tectum with anterior to the left; insets show the corresponding eye. (I,J) Schematic of the phenotypic outcome of *foxg1/foxd1* expression in the conditions shown in the figure. A-D are frontal views; G and H are lateral views of dissected eyes. All embryos are at 10-12ss except those in E-H, which are 6 dpf. Scale bars: 100 µm. Numbers in the bottom-right of each panel indicate the number of embryos with the phenotype shown out of the total number of embryos analysed. n*, defective nasal domain. Dashed lines outline the optic vesicles (A-D), the tectum (E,F) and the optic cup (insets in E,F).

The results described above indicated that Hh signalling is required to induce temporal identity at an early stage of optic vesicle development. To assess whether Hh activity is sufficient to promote *foxd1* expression and temporal identity, we expressed UAS:*shh* in the early, evaginating optic primordium by use of a Gal4 driver (*Tg{rx3:Gal4}*) expressed in the eye field and evaginating optic vesicles (Weiss et al., 2012). This approach did not interfere with the establishment of primary subdivisions in the forebrain, as revealed by the largely normal telencephalic expression of *foxg1* (compare Fig. 2A and 2B) and the optic vesicle and midbrain marker *mab21l2* (Fig. S2A,B).

Expression of Shh throughout the evaginating optic vesicles resulted in expansion of *foxd1* expression and repression of *foxg1* throughout the optic primordia but not in the adjacent telencephalon (Fig. 2A-D,I,J). The same effect within the optic vesicle was observed when we mosaically overexpressed Shh in subsets of eye field cells, or when Shh was overexpressed at low levels throughout the whole embryo (Fig. S3A-D′). Broad overexpression of *ptch2* confirmed that the exogenous Shh in these experiments ectopically activates the Hh pathway (Fig. S3E,G).

The enhanced expression of *foxd1* at the expense of *foxg1* in the presence of excessive Hh signalling suggested an expansion of temporal character in the optic primordium. To assess whether this change is reflected in the NT character of differentiated RGCs, we analysed the topology of retino-tectal projections by lipophilic dye labelling of nasal and temporal axons of wild-type and *Tg{rx3:Gal4}*;UAS:*shh* retinae. Nasal projections in wild-type 6 days post-fertilisation (dpf) fry innervated posterior regions of the tectum and clearly segregated from temporal projections (Fig. 2E). By contrast, nasal projections in *Tg{rx3::Gal4}*;UAS:*shh* retinae targeted more anterior regions of the tectum and partially overlapped with projections from the most temporal part of the retina (Fig. 2F). This suggests that nasally positioned RGCs acquire temporal identity after early exposure of the optic vesicle to Hh activity. This change of character is consistent with the widespread expansion of *foxd1* in retinal ganglion cells of *Tg{rx3::Gal4}*;UAS:*shh* embryos (Fig. 2G,H).

### Fgf does not appear to affect levels of Hh signalling whereas Hh activity promotes Fgf signalling

Previous studies have shown that Fgf signalling promotes nasal identity in the optic vesicles; abrogation of Fgf activity results in the loss of nasal identity and the concomitant expansion of temporal fate (Picker and Brand, 2005; Picker et al., 2009). Thus, whereas loss of Fgf activity results in a transformation of nasal into temporal identity, loss of Hh activity instead leads to a loss of temporal character that is not accompanied by acquisition of nasal character. A possible contributory factor to these phenotypes would be cross regulation of Hh and Fgf signalling pathways. Consequently, we analysed expression of *fgf8* and the Fgf pathway target *sprouty4* in Hh loss-of-function embryos, and that of *shh* and the Hh target transgene *ptch2::kaede* (Huang et al., 2012), after interference with Fgf signalling.

**Fig. 3.**
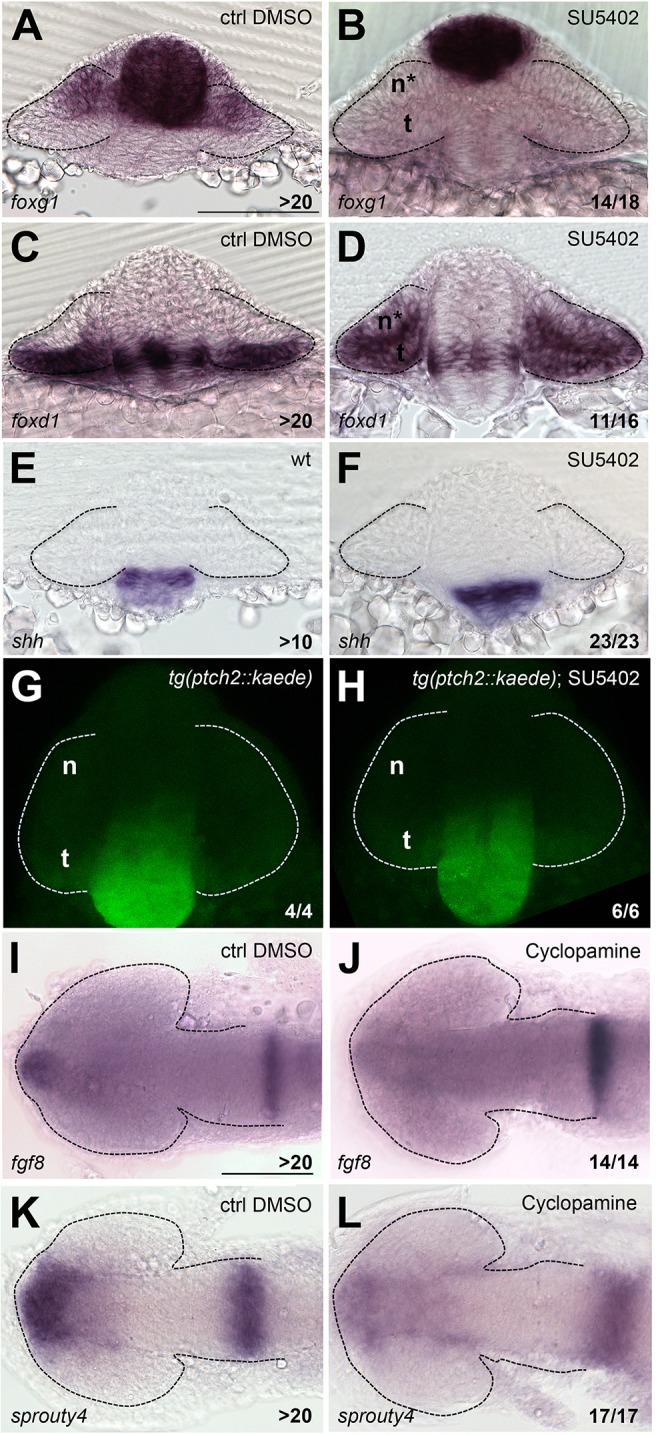
**Lack of Fgf activity alters NT patterning independently of Shh activity.** (A-L) Expression of *foxg1* (A,B), *foxd1* (C,D), *shh* (E,F), Kaede (G,H), *fgf8* (I,J) and *sprouty4* (K,L) in the conditions specified in the panels. A-H are frontal views; I-L are dorsal views with anterior to the left. All embryos are at 10-12ss. Scale bars: 100 µm. Numbers in the bottom-right of each panel indicate the number of embryos with the phenotype shown out of the total number of embryos analysed. n, nasal; t, temporal; n*, defective nasal domain. Dashed lines outline the forebrain (dorsal views) or the optic vesicles (frontal views).

Blocking Fgf signalling with the antagonist SU5402 (Mohammadi et al., 1997) from 1-2ss onwards efficiently transformed nasal to temporal character in the optic vesicle, as revealed by expanded *foxd1* and loss of *foxg1* expression (Fig. 3A-D; Picker et al., 2009). However, neither the expression of *shh* nor that of the *ptch2::GFP* transgene was affected by this treatment (Fig. 3E-H), suggesting that in the absence of Fgf signalling, Hh activity is largely unaffected.

Conversely, the level of Hh activity does affect Fgf signalling as cyclopamine treatments reduced the levels of both *fgf8* and *sprouty4* expression (Fig. 3I-L). This observation may help to explain why in the absence of Shh, nasal identity does not expand because there may be insufficient levels of inducer (Fgf) in the ventral portion of the optic vesicle to activate *foxg1*.

### Simultaneous abrogation of Fgf and Hh partially rescues NT patterning

Our results indicate that Hh activity is necessary and sufficient to promote *foxd1* expression in the optic vesicle. Furthermore, the observation that *foxg1* does not expand when Hh activity is downregulated suggests that nasal and temporal identities might be established independently from each other by Fgf and Hh signals, respectively. If so, one might expect that simultaneous abrogation of Fgf and Hh activity should then lead to the absence of both nasal and temporal character. To test this hypothesis, we simultaneously abrogated Fgfs and Hhs by making use of two different approaches: analysis of double mutants for *fgf8* (*acerebellar*; *ace*) and *smu*; and combined treatment with the pathway antagonists cyclopamine and SU5402.

**Fig. 4.**
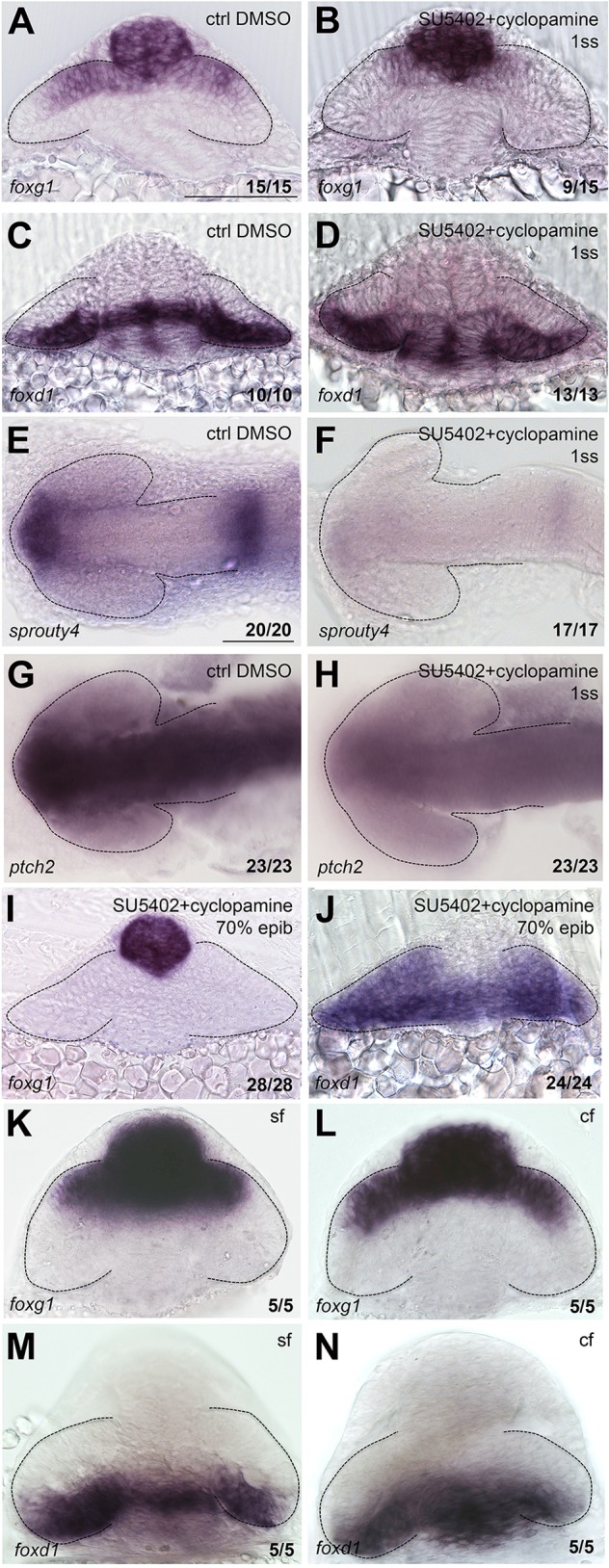
**NT patterning is restored upon combined abrogation of both Fgf and Hh signals.** Expression of *foxg1* (A,B,I,K,L), *foxd1* (C,D,J,M,N), *sprouty4* (E,F) and *ptch2* (G,H) in in the conditions specified in the panels. A-D,I-N are frontal views; E-H are dorsal views with anterior to the left. All are zebrafish embryos at 10-12ss, except for those in K-N, which are cavefish (cf) and surface fish (sf) forms of *Astyanax mexicanus*. Scale bars: 100 µm. Numbers in the bottom-right of each panel indicate the number of embryos with the phenotype shown out of the total number of embryos analysed. Dashed lines outline the forebrain (dorsal views) or the optic vesicles (frontal views).

Contrary to expectation, simultaneous abrogation of Hh and Fgf signals led to a surprising recovery of NT patterning. Thus, whereas cyclopamine treatment alone led to absence of *foxd1* expression (Fig. 1H,I), when combined with SU5402, expression of *foxd1* and the *HGn42A::GFP* transgene was restored within the prospective temporal retina (Fig. 4A-D; Fig. S1H). A similar result is observed in *ace*;*smu* double mutants (Fig. S4D,E). This implies that Hh signalling is only needed for induction of temporal character when Fgf signalling is active (and that Fgf activity represses *foxd1* independently of Foxg1). This result cannot be explained by a failure of the drugs to work when in combination as expression of the pathway reporters *ptch2* and *sprouty4* is largely lost following cyclopamine+SU5402 treatments (Fig. 4E-H). Thus, simultaneous loss of Hh and Fgf activity compromises NT patterning less than manipulation of just one of these signals, suggesting that NT patterning is influenced by the correct balance of both signals, and not by their absolute levels.

Similar to the observed restoration of temporal character, there was partial restoration of nasal *foxg1* expression upon abrogation of both Hh and Fgf signals (Fig. 4A,B; Fig. S1L; Fig. S4), which is more complete in *ace*;*smu* mutants than in fish treated with cyclopamine+SU5402 (compare Fig. 4B with Fig. S4D), as also confirmed by statistical analysis (Fig. S4A-C). This difference is probably due to the fact that in *ace*;*smu* double mutants Fgf abrogation is only partial, as the presence of Fgf3 and Fgf24 still probably activates the Fgf pathway (Picker et al., 2009). As expression of *foxg1* was not fully restored in the absence of both Fgf and Hh signals, it suggests a more important role for Fgf signals in promoting nasal character than Hh signals in promoting temporal character. This result, together with the fact that *foxg1* does not expand to the temporal retina in the absence of Hh (Fig. 1J,K), further reinforces the idea that Hh does not directly repress *foxg1*.

The abrogation of Fgf and Hh activities simultaneously from 1ss resulted, as shown above, in a partial recovery of the NT pattern. This suggests that earlier signalling events might be establishing *foxd1/foxg1* expression. To assess whether even earlier modulation of the Hh and Fgf signalling pathways affects the spatially restricted expression of *foxd1/foxg1* in the optic vesicle, we simultaneously abrogated Fgf and Hh signalling from mid-gastrulation, well before NT patterning is established.

Cyclopamine+SU5402 treatments from mid-gastrulation result in a dramatic expansion of *foxd1* and complete loss of *foxg1* expression within the optic vesicle (Fig. 4I,J), a phenotype comparable to that obtained by treatment with SU5402 alone [treatment from mid-gastrulation with only one drug at a time led to phenotypes very similar to those obtained with treatments at 1ss (not shown)]. This result supports the idea that Hh activity is fully dispensable for induction of *foxd1* expression in the absence of Fgf signalling. It suggests that Hh signalling prevents repression of *foxd1* by the Fgf signalling pathway, and, in this way, promotes temporal identity.

To further explore cross-regulation between Fgf and Hh pathways, we analysed NT patterning in cavefish (*Astyanax mexicanus*) embryos in which levels of Fgfs and *shh* vary between surface fish and cavefish forms. The species *Astyanax mexicanus* has a surface form, which lives in rivers and lakes, and a cavefish form, which lives in caves. These two populations were isolated from each other ∼10,000 years ago, and since then they have evolved divergently. The cavefish form has undergone a number of morphological changes in the forebrain, which seem to have their origin in subtle changes in expression patterns of regulatory genes during forebrain development (Pottin et al., 2011). One of these changes is an increased level of *shh* and precocious expression of *fgf8* in the forebrain of the cavefish form in comparison to the surface fish form. Thus, cavefish present the opportunity to assess the effect of contemporaneously higher levels of *fgf8* and *shh* on NT patterning of the optic vesicles. We reasoned that if Shh counteracts the repressive activity of Fgfs upon *foxd1* expression, then higher levels of both signals may not compromise NT patterning. Indeed, cavefish optic vesicles show similar levels of *foxg1* and *foxd1* expression compared with surface fish (Fig. 4K-N), indicating that concomitant upregulation of the Hh and Fgf pathways does not overtly affect NT patterning. Together, these results support the idea that it is the relative, rather than the absolute, levels of these two signals that influence the establishment of NT identity.

### Mutual repression between *foxg1* and *foxd1* maintains the border between nasal and temporal domains

Our results indicate that Fgf and Hh signals work in concert to promote mutually exclusive expression of *foxg1* and *foxd1* in the nasal and temporal retina, respectively. Previous studies in chick and mouse suggest that *foxd1* and *foxg1* can repress each other. For example, *Foxd1* expression expands into the nasal half of the optic vesicle in *Foxg1* mouse mutants (Huh et al., 1999), and misexpression of *Foxd1* or *Foxg1* interferes with the expression of the complementary gene in chick (Takahashi et al., 2009, 2003). To assess whether Foxg1 and Foxd1 cross-repress each other in zebrafish, we manipulated the levels of *foxg1* and *foxd1* in the optic vesicle through use of the Gal4/UAS approach as described above.

**Fig. 5.**
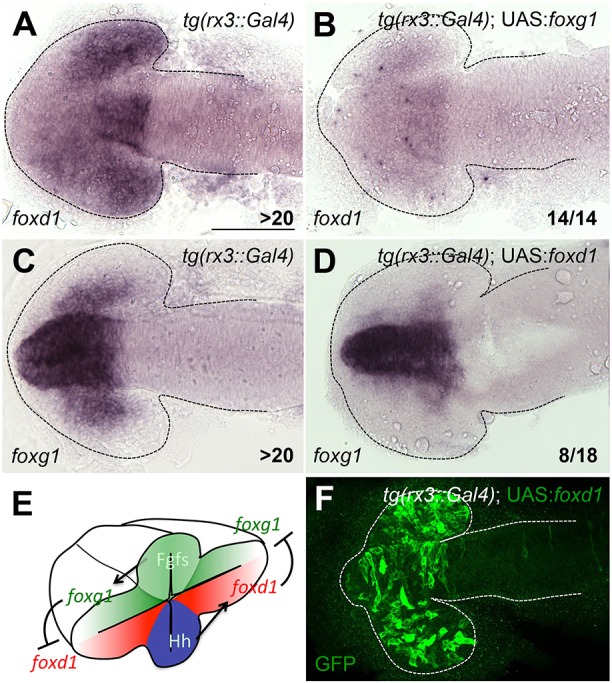
**Mutual repression between *foxg1* and *foxd1* maintains the NT border.** (A-D) *foxd1* (A,B) and *foxg1* (C,D) expression in the conditions detailed in the panels. All panels show dorsal views with anterior to the left at 10-12ss. (E) Schematic representation of the regulatory interactions inferred from our manipulations. (F) Representative *Tg (rx3:Gal4)*;* UAS:foxd1* embryo showing widespread GFP expression in the optic vesicles. All embryos selected for *in situ* analysis showed similarly broad GFP expression. Scale bar: 100 µm. Numbers in the bottom-right of each panel indicate the number of embryos with the phenotype shown out of the total number of embryos analysed. Dashed lines outline the forebrain.

Ectopic expression of *foxg1* in the temporal half of the optic vesicle strongly downregulated *foxd1* (Fig. 5A,B); conversely, *foxd1* expression in the nasal part of the optic vesicle downregulated *foxg1* expression (Fig. 5C,D). Thus, reciprocal repression between *foxg1* and *foxd1* occurs in fish as in other vertebrates. During normal development, the only position at which transcriptional cross-regulatory competition between Foxd1 and Foxg1 is likely to influence *foxg1* and *foxd1* expression is around the NT boundary where cells may receive sufficient Shh and Fgf signals to induce both genes.

## DISCUSSION

**Fig. 6.**
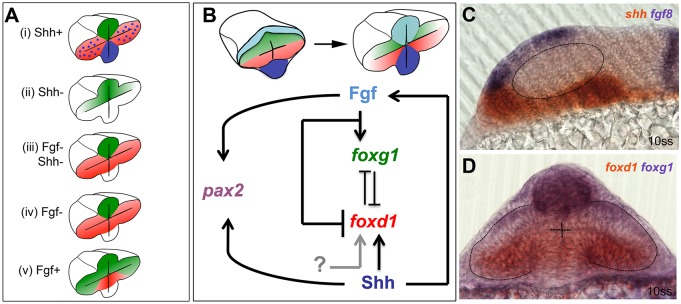
**Opposing roles for Fgfs and Shh in the control of optic vesicle patterning.** (A) Schematics of *foxg1* (green) and *foxd1* (red) expression in optic vesicles following manipulations of Fgf and Hh signals. The implications below are based on the ability of Foxg1 to repress *foxd1* expression and Foxd1 to repress *foxg1* expression. (i) Shh gain of function: loss of *foxg1* and gain of *foxd1* in nasal retina. This implies that Shh signalling promotes *foxd1* expression and/or inhibits *foxg1* expression. Blue shading and dots represent Shh expression. (ii) Shh loss of function: loss of *foxd1* in temporal retina. This implies that Shh promotes *foxd1* expression but is not required for repression of *foxg1*. (iii) Combined loss of Shh and Fgf: loss of *foxg1* and gain of *foxd1* in nasal retina. This implies that either unknown signals (grey arrow in B) promote *foxd1* expression in absence of Shh or that repressors (such as Fgf itself) are removed in this situation. The result also implies that Fgf is required for the repression of *foxd1* in temporal retina (shown in ii), and that this repression is independent of Foxg1 (which is not expressed in temporal retina). (iv) Loss of Fgf expression: loss of *foxg1* and gain of *foxd1* in nasal retina. This implies that Fgf promotes *foxg1* and/or inhibits *foxd1* in nasal retina. (v) Gain of Fgf function: gain of *foxg1* expression and loss of *foxd1* expression in temporal retina (data taken from Picker and Brand, 2005; Picker et al., 2009). This implies that Fgf promotes *foxg1* expression and/or inhibits *foxd1* expression. (B) Proposed regulatory interactions that could explain the retinal nasotemporal phenotypes shown in A, together with data not shown that both Fgf and Shh promote development of *pax2*+ optic stalk identity in the proximal optic vesicle. As stated in the main text, the regulatory interactions leading to nasotemporal patterning occur from neural plate stage onwards. (C,D) Images showing the domains of expression in the forebrain of genes encoding the signals studied (C) and their Fox gene targets (D), as evident from double *in situ* hybridisation assays of 10ss embryos. Dashed lines outline the optic vesicles.

This study uncovers a novel role for Shh in initiating the expression of the temporal fate determinant *foxd1* in the ventral half of the evaginating optic vesicles. Consequently, an interplay between Hh signals and Fgfs, which promote *foxg1* expression in the dorsal, prospective nasal half of the optic vesicle, establishes NT pattern in the nascent optic primordium. Our results indicate that these two signals establish temporal and nasal identity at least in part independently of each other and that, once established, the boundary between nasal and temporal domains is maintained by mutual transcriptional repression between Foxd1 and Foxg1 (Fig. 6).

### Similarities to, and differences from, other patterning systems involving Fgfs and Shh

The role for Shh and Fgfs that we describe for NT patterning of the optic vesicle is similar to that for anterior-posterior (AP) patterning of the otic vesicle, the primordium for the vertebrate ear. Fgfs, expressed rostral to the otic vesicle, promote anterior identity, whereas Shh, released by the tissues underlying the ear primordium, induces posterior identity (Hammond et al., 2003, 2010; Hammond and Whitfield, 2011). Manipulation of the levels of these two pathways affect AP patterning in the otic vesicle in a reciprocal way: loss of Fgf activity results in loss of anterior identity and the development of a double-posterior primordium; conversely, loss of Hh activity results in loss of posterior identity and the development of a partial double-anterior primordium. However, double loss of Hh and Fgfs results in an otic vesicle with neither anterior nor posterior identities, whereas in the optic vesicles NT patterning is partially recovered in such conditions.

Loss of both Shh and Fgf from mid-gastrula stage leads to absence of *foxg1* expression, indicating that Fgf activity from gastrula stages onwards promotes subsequent expression of *foxg1* expression in the prospective nasal retina. In addition, in these conditions *foxd1* expression expands throughout the optic vesicle, reinforcing the idea that Shh is dispensable for *foxd1* expression, provided there is no Fgf activity. Thus, acquisition of temporal identity normally requires the activity of Shh from as early as neural plate stages, to counteract Fgf-dependent repression of *foxd1* expression.

The recovery of *foxd1* expression in the optic vesicle in conditions in which both Fgf and Shh are abrogated is not the only situation in which loss of Hh activity can be compensated by following additional genetic changes. In the spinal cord, graded responses to Shh establish ventral neuronal identities and, consequently, ventral fates are lost upon removal of Shh activity (reviewed by Cohen et al., 2014; Dessaud et al., 2008). Ventral identities are, however, largely recovered when the function of the Gli3 transcriptional repressor of Hh target genes is also removed (Persson et al., 2002). Thus, acquisition of ventral spinal cord cell type identities can occur in a Shh-independent mechanism. This reveals a remarkable robustness in the establishment of DV patterning in the neural tube and NT patterning in the optic vesicle, and suggests the presence of compensatory mechanisms that can bypass requirement for Hh signalling.

A surprising aspect of the retinal NT phenotype following abrogation of both Shh and Fgf is the implication that Fgf is required for repression of *foxd1* expression in the temporal retina independently of Foxg1 (and in addition to the Fgf-dependent repression of *foxd1* in nasal retina that could be mediated through Foxg1; Fig. 5A,B). At least at the stages when optic vesicles initiate Fox gene expression, Fgf targets do not appear to be expressed in the prospective temporal domain (Picker et al., 2009; M.H.-B., F.C., G.G. and S.W.W., unpublished observations). This implies either that the Fgf pathway is activated earlier in this domain, or, if at the stage when Fox genes are induced, at sufficiently low levels so as to not activate expression of *foxg1*. An alternative possibility is that the repression is indirect and dependent upon non-autonomous consequences of Fgf activity in nasal retina. Although again we do not know how this might occur, Gli protein regulation is a likely target for regulation of the Hh pathway given that Gli function can be modulated by other pathways in a variety of other contexts (Aberger and Ruiz i Altaba, 2014).

Despite our results showing limited transcriptional cross-regulation between Shh and Fgf signalling during NT patterning of the optic vesicle, these pathways show many such regulatory interactions in other contexts. For example, Shh promotes *fgf8* expression in the rostral-most tip of the prosencephalon, and Fgf in turn promotes basal telencephalic *Shh* expression in a cross-regulatory interaction that modulates telencephalic patterning (Aoto et al., 2002; Danesin et al., 2009; Ohkubo et al., 2002; Shanmugalingam et al., 2000; Shinya et al., 2001; Storm et al., 2006; Walshe and Mason, 2003; this study). Similarly in cavefish, enhanced levels of *shh* expression at neural plate stages is correlated with precocious and stronger expression of *fgf8* in the prospective telencephalon (Menuet et al., 2007; Pottin et al., 2011). In the limb, Shh (expressed in the posterior portion of the primordium, known as the zone of polarising activity) and Fgfs (expressed in the distal portion of the limb primordium, termed the apical ectodermal ridge) engage in a complex regulatory feedback loop essential for allocation of correct proportions to elements in the growing limb (reviewed by Benazet et al., 2009; Benazet and Zeller, 2009; Scherz et al., 2004; Zuniga et al., 1999). In the ventral CNS, coordinated Fgf and Shh activities regulate the generation of cell diversity (Sasai et al., 2014). In this context, spatiotemporal coincidence of Shh and Fgf signalling in the caudal neural tube provides temporally constrained competence to initiate floor plate specification. As the neural tube extends, the source of Fgf is distanced from the ventral spinal cord and Shh acts independently to promote ventral neuronal fates.

### Fgf and Shh signals pattern both the NT and DV axes of the optic vesicles

In addition to roles in NT patterning, the Fgf and Hh signalling pathways are also required for formation of proximal optic stalk fates within the optic vesicle. Both Fgfs and *shh* are expressed in the anterior-most tip of the forebrain, adjacent to the region at which the optic vesicles remain connected to the forebrain through the optic stalks. As previously shown, alterations to either signalling pathway can shift the optic stalk/retina boundary and disrupt optic stalk/nerve differentiation (Cardozo et al., 2014; Chiang et al., 1996; Ekker et al., 1995; Lupo et al., 2005; Macdonald et al., 1995; Martinez-Morales et al., 2005; Perron et al., 2003; Take-uchi et al., 2003; Walshe and Mason, 2003). We propose that the specific outcomes of the activity of these two pathways on the forming eye are the consequence of the differing spatial distributions of signals coupled with temporally regulated receptiveness of optic vesicle cells as they undergo dynamic morphogenetic movements (see model in Fig. 6; Picker et al., 2009). Indeed, *shh* and Fgfs are expressed in adjacent domains at the anterior-most region of the forebrain, and thus the anterior-most region of the evaginating optic vesicles – the presumptive optic stalk – is probably exposed to both signals. More posteriorly, as optic vesicle cells evaginate into the prospective temporal retina, they are probably exposed to Hh signals alone whereas as they ingress into the prospective nasal retina they are exposed to Fgf signals.

Eye field cells extensively intercalate among each other as they incorporate in the evaginating primordia (Ivanovitch et al., 2013). We have speculated that this mixing means that it is not possible to predict the final fate of many cells within the eye field and, consequently, we have proposed that regional fate would only be established after cells have evaginated into the optic vesicles. The results shown in this study show that signals influencing NT patterning are acting from very early stages, probably prior to completion of the integration of eye field cells into the optic vesicles. However, the signals required to establish NT pattern are produced and secreted by dorsal and ventral forebrain territories with organiser-like properties (Picker et al., 2009). These territories constitute ‘fixed’ domains relative to the eye field/optic vesicle, and exert their influence upon cells entering either the dorsal or the ventral half of the eye primordium, irrespective of their original location within the eye field prior to evagination. This mechanism of fixing the sources of signals could provide robustness to patterning in morphogenetic contexts where cells are undergoing dynamic reorganisations.

### Generating sharp boundaries downstream of morphogenetic signals

In the spinal cord, Shh controls the expression of transcription factors that collectively subdivide the neural tube into discrete generative domains along its DV axis (reviewed by Dessaud et al., 2008). Shh-regulated transcription factor-encoding genes expressed in adjacent domains are frequently cross-repressive. This has the consequence that any individual cell (usually at a boundary between domains) would resolve its expression to one or other of the mutually repressive genes, thereby sharpening the boundary between domains (Cohen et al., 2013). The scenario we describe in the optic vesicle is highly reminiscent of this mode of patterning. Shh initiates expression of *foxd1*, and then Foxd1 represses *foxg1*, which is induced by Fgfs and, in turn, Foxg1 can repress *foxd1*. This cross-repression would then ensure that the cells at the NT boundary would only adopt either nasal or temporal identity. In this way, the early graded activity of Shh and Fgf could be translated into the establishment of a sharp NT border that is maintained throughout later stages of eye development.

## MATERIALS AND METHODS

### Fish lines and husbandry

*AB* and *tupl* wild-type zebrafish strains, and transgenic lines *Tg{rx3::GFP}^ET95/1^* (Brown et al., 2010; Rembold et al., 2006), *Tg{emx3::YFP}^b1200^* (Viktorin et al., 2009), *Tg{rx3::Gal4-VP16}^vu271Tg^* (Weiss et al., 2012), *Tg{ptch2::kaede}^a4596Tg^* (Huang et al., 2012), *Tg{-8.0claudinb::lynGFP}^zf106^* (Haas and Gilmour, 2006) and *Tg{HGn42A::GFP}^nkhgn42aEt^* (Picker et al., 2009) were maintained and bred according to standard procedures (Westerfield, 1993). Laboratory stocks of *A. mexicanus* surface fish and cavefish (Pachón population) were obtained from the Yamamoto laboratory at UCL. All experiments conform to the guidelines from the European Community Directive and the British and Spanish legislation for the experimental use of animals.

### Microinjection and drug treatments

*shh*, *foxd1* and *foxg1* were expressed in the optic vesicles using the UAS/Gal4 system (Halpern et al., 2008). UAS constructs were generated by subcloning the test cDNA into a bidirectional UAS/tol2 plasmid, which drives GFP transcription in one direction and the test cDNA transcription in the other (Distel et al., 2010; Kajita et al., 2014). UAS constructs were injected into one-cell-stage *Tg{rx3::Gal4}* embryos (at 20-40 pg/embryo) and the embryos showing homogeneous GFP expression in the eye primordia were selected for further analysis. *shh* mRNA for microinjection was synthesised using the mMessage Machine kit (Ambion), following the manufacturer's instructions.

Drug treatments were performed by incubating dechorionated embryos in E3 medium with cyclopamine (100 μM, Calbiochem), SU5402 (10 μM, Calbiochem) or a combination of both. As stocks of cyclopamine and SU5402 were kept in DMSO, controls for these treatments were incubated in the same amount of E3 medium with the equivalent concentration of DMSO. The treatment was stopped at 10/12ss and the embryos were washed and fixed for further analysis. Note that in our experimental conditions, Shh/Fgf abrogations are performed once the first stages of forebrain patterning have taken place, and thus are unlikely to promote changes in primary forebrain subdivisions, as revealed by the normal expression of optic vesicle and telencephalic markers (Fig. S2C-H; see also Rohr et al., 2001; Shinya et al., 2001).

### mRNA detection and immunolabelling

Antisense mRNA probes for whole-mount *in situ* hybridisation were synthesised using RNA polymerases (Promega) and digoxigenin- or fluorescein-labelled nucleotides (Roche), following the manufacturer's instructions. Whole-mount *in situ* hybridisations were performed essentially as previously described (Cavodeassi et al., 2013; Yamamoto et al., 2004). For visualisation, embryos were incubated with anti-digoxigenin/fluorescein-AP and developed using NBT/BCIP substrates (Roche). For fluorescent detection, embryos were incubated with anti-digoxigenin-POD (Roche) and developed using Cy3-TSA (Perkin Elmer) as a substrate. Immunolabelling was performed as previously described (Cavodeassi et al., 2013) with the following antibodies: chicken anti-GFP (Abcam, cat. no. ab13970; 1:1000); mouse anti-βcatenin (Signal Transduction Laboratories, cat no. 610154; 1:400) and Alexa-488 and -647 coupled secondary antibodies (Jackson ImmunoResearch, 1:500). Sytox Orange (Life Technologies, 1:10,000) was used to counterstain nuclei.

### Tracing of retinotectal projections

DiI and DiO were used to label nasal and temporal retinal ganglion cells at 6 dpf in paraformaldehyde-fixed wild-type and *Tg{rx3::Gal4}*; UAS:*Shh* retinae. Fry were incubated at room temperature for 24 h before preparing them for imaging. Each tectum and its corresponding eye were sequentially imaged.

### Imaging and data processing

DiI/DiO-traced embryos and *Tg{ptch2::kaede}^a4596Tg^* embryos were embedded in low melting point agarose (Sigma) at 1-1.5% in PBS for confocal imaging using a 40× (0.8NA) long-working distance water immersion lens. A Zeiss LSM710 confocal microscopy system was used for image acquisition.

*In situ* hybridised embryos were mounted flat in a drop of glycerol and dorsal images were acquired with a 20× (0.70NA) dry lens using a Leica CTR 5000 microscope connected to a digital camera (Leica DFC 500), and operated by Leica software. Some of these embryos were embedded in gelatine/BSA for vibratome sectioning as previously described (Sanchez-Arrones et al., 2013). Sections (20 μm thick) were obtained using a Leica VT1000S vibratome, mounted in glycerol, and imaged with a 40× (0.85NA) dry lens.

Raw confocal data were analysed with Fiji/ImageJ. Images were exported as TIFF files and all figures were composed using Photoshop.

## References

[DEV125120C1] Aberger F. and Ruiz i Altaba A. (2014). Context-dependent signal integration by the GLI code: the oncogenic load, pathways, modifiers and implications for cancer therapy. *Semin. Cell Dev. Biol.* 33, 93-104. 10.1016/j.semcdb.2014.05.003 24852887 PMC4151135

[DEV125120C2] Aoto K., Nishimura T., Eto K. and Motoyama J. (2002). Mouse GLI3 regulates Fgf8 expression and apoptosis in the developing neural tube, face, and limb bud. *Dev. Biol.* 251, 320-332. 10.1006/dbio.2002.0811 12435361

[DEV125120C3] Barth K. A. and Wilson S. W. (1995). Expression of zebrafish nk2.2 is influenced by sonic hedgehog/vertebrate hedgehog-1 and demarcates a zone of neuronal differentiation in the embryonic forebrain. *Development* 121, 1755-1768. 7600991 10.1242/dev.121.6.1755

[DEV125120C4] Benazet J.-D. and Zeller R. (2009). Vertebrate limb development: moving from classical morphogen gradients to an integrated 4-dimensional patterning system. *Cold Spring Harb. Perspect. Biol.* 1, a001339 10.1101/cshperspect.a001339 20066096 PMC2773624

[DEV125120C5] Benazet J.-D., Bischofberger M., Tiecke E., Goncalves A., Martin J. F., Zuniga A., Naef F. and Zeller R. (2009). A self-regulatory system of interlinked signaling feedback loops controls mouse limb patterning. *Science* 323, 1050-1053. 10.1126/science.1168755 19229034

[DEV125120C6] Bourguignon C., Li J. and Papalopulu N. (1998). XBF-1, a winged helix transcription factor with dual activity, has a role in positioning neurogenesis in Xenopus competent ectoderm. *Development* 125, 4889-4900. 9811573 10.1242/dev.125.24.4889

[DEV125120C7] Briscoe J. and Therond P. P. (2013). The mechanisms of Hedgehog signalling and its roles in development and disease. *Nat. Rev. Mol. Cell Biol.* 14, 416-429. 10.1038/nrm3598 23719536

[DEV125120C8] Brown K. E., Keller P. J., Ramialison M., Rembold M., Stelzer E. H. K., Loosli F. and Wittbrodt J. (2010). Nlcam modulates midline convergence during anterior neural plate morphogenesis. *Dev. Biol.* 339, 14-25. 10.1016/j.ydbio.2009.12.003 20005219

[DEV125120C9] Cardozo M. J., Sánchez-Arrones L., Sandonis A., Sánchez-Camacho C., Gestri G., Wilson S. W., Guerrero I. and Bovolenta P. (2014). Cdon acts as a Hedgehog decoy receptor during proximal-distal patterning of the optic vesicle. *Nat. Commun.* 5, 4272 10.1038/ncomms5272 25001599 PMC4102123

[DEV125120C10] Carreres M. I., Escalante A., Murillo B., Chauvin G., Gaspar P., Vegar C. and Herrera E. (2011). Transcription factor Foxd1 is required for the specification of the temporal retina in mammals. *J. Neurosci.* 31, 5673-5681. 10.1523/JNEUROSCI.0394-11.2011 21490208 PMC6622810

[DEV125120C11] Cavodeassi F., Ivanovitch K. and Wilson S. W. (2013). Eph/Ephrin signalling maintains eye field segregation from adjacent neural plate territories during forebrain morphogenesis. *Development* 140, 4193-4202. 10.1242/dev.097048 24026122 PMC3787759

[DEV125120C12] Chen J. K., Taipale J., Cooper M. K. and Beachy P. A. (2002). Inhibition of Hedgehog signaling by direct binding of cyclopamine to Smoothened. *Genes Dev.* 16, 2743-2748. 10.1101/gad.1025302 12414725 PMC187469

[DEV125120C13] Chiang C., Litingtung Y., Lee E., Young K. E., Corden J. L., Westphal H. and Beachy P. A. (1996). Cyclopia and defective axial patterning in mice lacking Sonic hedgehog gene function. *Nature* 383, 407-413. 10.1038/383407a0 8837770

[DEV125120C14] Cobos I., Shimamura K., Rubenstein J. L. R., Martínez S. and Puelles L. (2001). Fate map of the avian anterior forebrain at the four-somite stage, based on the analysis of quail-chick chimeras. *Dev. Biol.* 239, 46-67. 10.1006/dbio.2001.0423 11784018

[DEV125120C15] Cohen M., Briscoe J. and Blassberg R. (2013). Morphogen interpretation: the transcriptional logic of neural tube patterning. *Curr. Opin. Genet. Dev.* 23, 423-428. 10.1016/j.gde.2013.04.003 23725799

[DEV125120C16] Cohen M., Page K. M., Perez-Carrasco R., Barnes C. P. and Briscoe J. (2014). A theoretical framework for the regulation of Shh morphogen-controlled gene expression. *Development* 141, 3868-3878. 10.1242/dev.112573 25294939 PMC4197706

[DEV125120C17] Danesin C., Peres J. N., Johansson M., Snowden V., Cording A., Papalopulu N. and Houart C. (2009). Integration of telencephalic Wnt and hedgehog signaling center activities by Foxg1. *Dev. Cell* 16, 576-587. 10.1016/j.devcel.2009.03.007 19386266

[DEV125120C18] Dessaud E., McMahon A. P. and Briscoe J. (2008). Pattern formation in the vertebrate neural tube: a sonic hedgehog morphogen-regulated transcriptional network. *Development* 135, 2489-2503. 10.1242/dev.009324 18621990

[DEV125120C19] Distel M., Hocking J. C., Volkmann K. and Koster R. W. (2010). The centrosome neither persistently leads migration nor determines the site of axonogenesis in migrating neurons in vivo. *J. Cell Biol.* 191, 875-890. 10.1083/jcb.201004154 21059852 PMC2983064

[DEV125120C20] Ekker S. C., Ungar A. R., Greenstein P., von Kessler D. P., Porter J. A., Moon R. T. and Beachy P. A. (1995). Patterning activities of vertebrate hedgehog proteins in the developing eye and brain. *Curr. Biol.* 5, 944-955. 10.1016/S0960-9822(95)00185-0 7583153

[DEV125120C21] Erskine L. and Herrera E. (2007). The retinal ganglion cell axon's journey: insights into molecular mechanisms of axon guidance. *Dev. Biol.* 308, 1-14. 10.1016/j.ydbio.2007.05.013 17560562

[DEV125120C22] Haas P. and Gilmour D. (2006). Chemokine signaling mediates self-organizing tissue migration in the zebrafish lateral line. *Dev. Cell* 10, 673-680. 10.1016/j.devcel.2006.02.019 16678780

[DEV125120C23] Halpern M. E., Rhee J., Goll M. G., Akitake C. M., Parsons M. and Leach S. D. (2008). Gal4/UAS transgenic tools and their application to zebrafish. *Zebrafish* 5, 97-110. 10.1089/zeb.2008.0530 18554173 PMC6469517

[DEV125120C24] Hammond K. L. and Whitfield T. T. (2011). Fgf and Hh signalling act on a symmetrical pre-pattern to specify anterior and posterior identity in the zebrafish otic placode and vesicle. *Development* 138, 3977-3987. 10.1242/dev.066639 21831919 PMC3160093

[DEV125120C25] Hammond K. L., Loynes H. E., Folarin A. A., Smith J. and Whitfield T. T. (2003). Hedgehog signalling is required for correct anteroposterior patterning of the zebrafish otic vesicle. *Development* 130, 1403-1417. 10.1242/dev.00360 12588855

[DEV125120C26] Hammond K. L., van Eeden F. J. M. and Whitfield T. T. (2010). Repression of Hedgehog signalling is required for the acquisition of dorsolateral cell fates in the zebrafish otic vesicle. *Development* 137, 1361-1371. 10.1242/dev.045666 20223756 PMC2847469

[DEV125120C27] Hardcastle Z. and Papalopulu N. (2000). Distinct effects of XBF-1 in regulating the cell cycle inhibitor p27(XIC1) and imparting a neural fate. *Development* 127, 1303-1314. 10683182 10.1242/dev.127.6.1303

[DEV125120C28] Hatini V., Tao W. and Lai E. (1994). Expression of winged helix genes, BF-1 and BF-2, define adjacent domains within the developing forebrain and retina. *J. Neurobiol.* 25, 1293-1309. 10.1002/neu.480251010 7815060

[DEV125120C29] Herrera E., Marcus R., Li S., Williams S. E., Erskine L., Lai E. and Mason C. (2004). Foxd1 is required for proper formation of the optic chiasm. *Development* 131, 5727-5739. 10.1242/dev.01431 15509772

[DEV125120C30] Huang P., Xiong F., Megason S. G. and Schier A. F. (2012). Attenuation of Notch and Hedgehog signaling is required for fate specification in the spinal cord. *PLoS Genet.* 8, e1002762 10.1371/journal.pgen.1002762 22685423 PMC3369957

[DEV125120C31] Huh S., Hatini V., Marcus R. C., Li S. C. and Lai E. (1999). Dorsal-ventral patterning defects in the eye of BF-1-deficient mice associated with a restricted loss of shh expression. *Dev. Biol.* 211, 53-63. 10.1006/dbio.1999.9303 10373304

[DEV125120C32] Ivanovitch K., Cavodeassi F. and Wilson S. W. (2013). Precocious acquisition of neuroepithelial character in the eye field underlies the onset of eye morphogenesis. *Dev. Cell* 27, 293-305. 10.1016/j.devcel.2013.09.023 24209576 PMC3898423

[DEV125120C33] Kajita M., Sugimura K., Ohoka A., Burden J., Suganuma H., Ikegawa M., Shimada T., Kitamura T., Shindoh M., Ishikawa S. et al. (2014). Filamin acts as a key regulator in epithelial defence against transformed cells. *Nat. Commun.* 5, 4428 10.1038/ncomms5428 25079702

[DEV125120C35] Lupo G., Liu Y., Qiu R., Chandraratna R. A. S., Barsacchi G., He R.-Q. and Harris W. A. (2005). Dorsoventral patterning of the Xenopus eye: a collaboration of Retinoid, Hedgehog and FGF receptor signaling. *Development* 132, 1737-1748. 10.1242/dev.01726 15753216

[DEV125120C36] Macdonald R., Barth K. A., Xu Q., Holder N., Mikkola I. and Wilson S. W. (1995). Midline signalling is required for Pax gene regulation and patterning of the eyes. *Development* 121, 3267-3278. 7588061 10.1242/dev.121.10.3267

[DEV125120C37] Martinez-Morales J.-R., Del Bene F., Nica G., Hammerschmidt M., Bovolenta P. and Wittbrodt J. (2005). Differentiation of the vertebrate retina is coordinated by an FGF signaling center. *Dev. Cell* 8, 565-574. 10.1016/j.devcel.2005.01.022 15809038

[DEV125120C38] Martynoga B., Morrison H., Price D. J. and Mason J. O. (2005). Foxg1 is required for specification of ventral telencephalon and region-specific regulation of dorsal telencephalic precursor proliferation and apoptosis. *Dev. Biol.* 283, 113-127. 10.1016/j.ydbio.2005.04.005 15893304

[DEV125120C39] Menuet A., Alunni A., Joly J.-S., Jeffery W. R. and Retaux S. (2007). Expanded expression of Sonic Hedgehog in Astyanax cavefish: multiple consequences on forebrain development and evolution. *Development* 134, 845-855. 10.1242/dev.02780 17251267

[DEV125120C40] Mohammadi M., McMahon G., Sun L., Tang C., Hirth P., Yeh B. K., Hubbard S. R. and Schlessinger J. (1997). Structures of the tyrosine kinase domain of fibroblast growth factor receptor in complex with inhibitors. *Science* 276, 955-960. 10.1126/science.276.5314.955 9139660

[DEV125120C41] Ohkubo Y., Chiang C. and Rubenstein J. L. R. (2002). Coordinate regulation and synergistic actions of BMP4, SHH and FGF8 in the rostral prosencephalon regulate morphogenesis of the telencephalic and optic vesicles. *Neuroscience* 111, 1-17. 10.1016/S0306-4522(01)00616-9 11955708

[DEV125120C42] Perron M., Boy S., Amato M. A., Viczian A., Koebernick K., Pieler T. and Harris W. A. (2003). A novel function for Hedgehog signalling in retinal pigment epithelium differentiation. *Development* 130, 1565-1577. 10.1242/dev.00391 12620982

[DEV125120C43] Persson M., Stamataki D., te Welscher P., Andersson E., Bose J., Ruther U., Ericson J. and Briscoe J. (2002). Dorsal-ventral patterning of the spinal cord requires Gli3 transcriptional repressor activity. *Genes Dev.* 16, 2865-2878. 10.1101/gad.243402 12435629 PMC187477

[DEV125120C44] Picker A. and Brand M. (2005). Fgf signals from a novel signaling center determine axial patterning of the prospective neural retina. *Development* 132, 4951-4962. 10.1242/dev.02071 16236770

[DEV125120C45] Picker A., Cavodeassi F., Machate A., Bernauer S., Hans S., Abe G., Kawakami K., Wilson S. W. and Brand M. (2009). Dynamic coupling of pattern formation and morphogenesis in the developing vertebrate retina. *PLoS Biol.* 7, e1000214 10.1371/journal.pbio.1000214 19823566 PMC2751823

[DEV125120C46] Pottin K., Hinaux H. and Retaux S. (2011). Restoring eye size in Astyanax mexicanus blind cavefish embryos through modulation of the Shh and Fgf8 forebrain organising centres. *Development* 138, 2467-2476. 10.1242/dev.054106 21610028

[DEV125120C47] Rembold M., Lahiri K., Foulkes N. S. and Wittbrodt J. (2006). Transgenesis in fish: efficient selection of transgenic fish by co-injection with a fluorescent reporter construct. *Nat. Protoc.* 1, 1133-1139. 10.1038/nprot.2006.165 17406394

[DEV125120C48] Rohr K. B., Barth K. A., Varga Z. M. and Wilson S. W. (2001). The nodal pathway acts upstream of hedgehog signaling to specify ventral telencephalic identity. *Neuron* 29, 341-351. 10.1016/S0896-6273(01)00210-0 11239427

[DEV125120C49] Sanchez-Arrones L., Nieto-Lopez F., Sanchez-Camacho C., Carreres M. I., Herrera E., Okada A. and Bovolenta P. (2013). Shh/Boc signaling is required for sustained generation of ipsilateral projecting ganglion cells in the mouse retina. *J. Neurosci.* 33, 8596-8607. 10.1523/JNEUROSCI.2083-12.2013 23678105 PMC3827538

[DEV125120C50] Sasai N., Kutejova E. and Briscoe J. (2014). Integration of signals along orthogonal axes of the vertebrate neural tube controls progenitor competence and increases cell diversity. *PLoS Biol.* 12, e1001907 10.1371/journal.pbio.1001907 25026549 PMC4098999

[DEV125120C51] Schauerte H. E., van Eeden F. J., Fricke C., Odenthal J., Strahle U. and Haffter P. (1998). Sonic hedgehog is not required for the induction of medial floor plate cells in the zebrafish. *Development* 125, 2983-2993. 9655820 10.1242/dev.125.15.2983

[DEV125120C52] Scherz P. J., Harfe B. D., McMahon A. P. and Tabin C. J. (2004). The limb bud Shh-Fgf feedback loop is terminated by expansion of former ZPA cells. *Science* 305, 396-399. 10.1126/science.1096966 15256670

[DEV125120C53] Schulte D. and Bumsted-O'Brien K. M. (2008). Molecular mechanisms of vertebrate retina development: implications for ganglion cell and photoreceptor patterning. *Brain Res.* 1192, 151-164. 10.1016/j.brainres.2007.04.079 17553468

[DEV125120C54] Shanmugalingam S., Houart C., Picker A., Reifers F., Macdonald R., Barth A., Griffin K., Brand M. and Wilson S. W. (2000). Ace/Fgf8 is required for forebrain commissure formation and patterning of the telencephalon. *Development* 127, 2549-2561. 10821754 10.1242/dev.127.12.2549

[DEV125120C55] Shinya M., Koshida S., Sawada A., Kuroiwa A. and Takeda H. (2001). Fgf signalling through MAPK cascade is required for development of the subpallial telencephalon in zebrafish embryos. *Development* 128, 4153-4164. 11684653 10.1242/dev.128.21.4153

[DEV125120C56] Storm E. E., Garel S., Borello U., Hebert J. M., Martinez S., McConnell S. K., Martin G. R. and Rubenstein J. L. R. (2006). Dose-dependent functions of Fgf8 in regulating telencephalic patterning centers. *Development* 133, 1831-1844. 10.1242/dev.02324 16613831

[DEV125120C57] Taipale J., Chen J. K., Cooper M. K., Wang B., Mann R. K., Milenkovic L., Scott M. P. and Beachy P. A. (2000). Effects of oncogenic mutations in Smoothened and Patched can be reversed by cyclopamine. *Nature* 406, 1005-1009. 10.1038/35023008 10984056

[DEV125120C58] Takahashi H., Shintani T., Sakuta H. and Noda M. (2003). CBF1 controls the retinotectal topographical map along the anteroposterior axis through multiple mechanisms. *Development* 130, 5203-5215. 10.1242/dev.00724 12954716

[DEV125120C59] Takahashi H., Sakuta H., Shintani T. and Noda M. (2009). Functional mode of FoxD1/CBF2 for the establishment of temporal retinal specificity in the developing chick retina. *Dev. Biol.* 331, 300-310. 10.1016/j.ydbio.2009.05.549 19450575

[DEV125120C60] Take-uchi M., Clarke J. D. W. and Wilson S. W. (2003). Hedgehog signalling maintains the optic stalk-retinal interface through the regulation of Vax gene activity. *Development* 130, 955-968. 10.1242/dev.00305 12538521

[DEV125120C61] Varga Z. M., Amores A., Lewis K. E., Yan Y. L., Postlethwait J. H., Eisen J. S. and Westerfield M. (2001). Zebrafish smoothened functions in ventral neural tube specification and axon tract formation. *Development* 128, 3497-3509. 11566855 10.1242/dev.128.18.3497

[DEV125120C62] Viktorin G., Chiuchitu C., Rissler M., Varga Z. M. and Westerfield M. (2009). Emx3 is required for the differentiation of dorsal telencephalic neurons. *Dev. Dyn.* 238, 1984-1998. 10.1002/dvdy.22031 19650145 PMC2975037

[DEV125120C63] Walshe J. and Mason I. (2003). Unique and combinatorial functions of Fgf3 and Fgf8 during zebrafish forebrain development. *Development* 130, 4337-4349. 10.1242/dev.00660 12900450

[DEV125120C64] Weiss O., Kaufman R., Michaeli N. and Inbal A. (2012). Abnormal vasculature interferes with optic fissure closure in lmo2 mutant zebrafish embryos. *Dev. Biol.* 369, 191-198. 10.1016/j.ydbio.2012.06.029 22819672

[DEV125120C65] Westerfield M. (1993). *The Zebrafish Book: A Guide for the Laboratory Use of the Zebrafish (Brachydanio rerio)*. Eugene: University of Oregon Press.

[DEV125120C66] Yamamoto Y., Stock D. W. and Jeffery W. R. (2004). Hedgehog signalling controls eye degeneration in blind cavefish. *Nature* 431, 844-847. 10.1038/nature02864 15483612

[DEV125120C67] Zuniga A., Haramis A.-P. G., McMahon A. P. and Zeller R. (1999). Signal relay by BMP antagonism controls the SHH/FGF4 feedback loop in vertebrate limb buds. *Nature* 401, 598-602. 10.1038/44157 10524628

